# The role of hypoxia-inducible factor-2 in digestive system cancers

**DOI:** 10.1038/cddis.2014.565

**Published:** 2015-01-15

**Authors:** J Zhao, F Du, G Shen, F Zheng, B Xu

**Affiliations:** 1 Department of Medical Oncology, Cancer Institute and Hospital, Chinese Academy of Medical Science, Peking Union Medical College, Beijing, China; 2 Affiliated Hospital of Qinghai University, High Altitude Medical Research Center, Xining, China

## Abstract

Hypoxia is an all but ubiquitous phenomenon in cancers. Two known hypoxia-inducible factors (HIFs), HIF-1*α* and HIF-2*α*, primarily mediate the transcriptional response to hypoxia. Despite the high homology between HIF-1*α* and HIF-2*α*, emerging evidence suggests differences between both molecules in terms of transcriptional targets as well as impact on multiple physiological pathways and tumorigenesis. To date, much progress has been made toward understanding the roles of HIF-2*α* in digestive system cancers. Indeed, HIF-2*α* has been shown to regulate multiple aspects of digestive system cancers, including cell proliferation, angiogenesis and apoptosis, metabolism, metastasis and resistance to chemotherapy. These findings make HIF-2*α* a critical regulator of this malignant phenotype. Here we summarize the function of HIF-2 during cancer development as well as its contribution to tumorigenesis in digestive system malignancies.

## Facts

- Hypoxia is a common phenomenon in digestive system cancers.
- HIF-2*α* is an essential mediator of the cellular oxygen-signaling pathway.
- HIF-2 plays important roles in digestive system cancers and upregulates genes involved in cell proliferation, angiogenesis and apoptosis, metabolism, metastasis and resistance to chemotherapy.
- HIF-2 inhibitors can suppress the expression of HIF-2 target genes to reduce angiogenesis and metastasis.

## Open questions

- Studies assessing the HIF-2*α* roles in digestive system cancers are scarce, with inconsistent outcomes. Therefore, the actual roles of HIF-2*α* are still unclear.
- The definite mechanisms of HIF-2 activity during digestive system cancers remain elusive. How does HIF-2 take part in tumorigenesis of digestive system cancers?
- The exploitation of HIF-2*α* inhibitors faces many challenges.

## Introduction to HIF-2*α*

**Figure 1:**
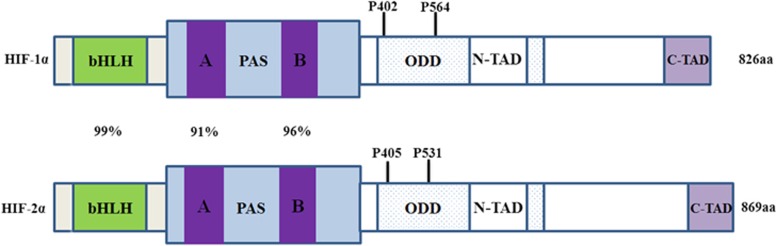
The structural domains of HIF-1*α* and HIF-2*α*. Both of them contain basic helix-loop-helix (bHLH), per-Arnt-SIM (PAS), oxygen-dependent degradation domain (ODD) and C-terminal transactivation domains (CADs). Prolyl hydroxylases (PHDs) hydroxylate proline residues 402 and 564, and 405 and 531, respectively, in ODD of HIF-1*α* and HIF-2*α*, under normoxic conditions, targeting it for degradation by the proteosome, respectively. Numbers refer to amino-acid similarity between human HIF-1*α* and HIF-2*α* in the defined domains

HIF-2 is the second member of the hypoxia-inducible factor (HIF) family that includes three proteins: HIF-1, HIF-2 and HIF-3. Its amino-acid sequence shares ∼48% homology with that of HIF-1.^1, 2, 3^ HIF-2 is a heterologous dimeric protein complex that consists of two subunits: HIF-2*α* and HIF-1*β*. Both HIF-2*α* and HIF-1*β* belong to the superfamily of transcription factors bHLH-PAS. HIF-2*α*, also named endothelial PAS domain protein-1 (EPAS1), member of pas superfamily 2 (Mop2), hypoxia-inducible factor 2*α* subunit or Hif-2-*α*, was identified in 1997.^1, 2, 3, 4^ It binds to and activates transcription from the HIF-1*α* response element derived from the 3-prime flanking region of the *EPO* gene. Hypoxic conditions stimulate HIF-2*α* activation.^5^ Like HIF-1, HIF-2 is primarily regulated by specific prolyl hydroxylase-domain enzymes (PHDs) that initiate its degradation via the von Hippel–Lindau protein (pVHL) tumor suppressor protein.^6^ Under normoxia, PHDs hydroxylate two conserved proline residues (Pro 405 and 531) within HIF-2*α*, using oxygen, *α*-ketoglutarate and iron as cofactors. HIF-2*α* hydroxylation facilitates the binding of pVHL to the HIF-2*α* ODD^7^ (Figure 1). The pVHL protein constitutes the substrate recognition module of an E3 ubiquitin ligase complex comprising elongin C, elongin B, cullin-2 and ring-box 1 that directs HIF-2*α* polyubiquitylation and proteasomal degradation.^8^ With the inhibition of PHD activity and elimination of pVHL binding under hypoxia, HIF-2*α* enters the nucleus and heterodimerizes with HIF-1*β*. Subsequently, HIF-2*α* binds to a conserved DNA sequence known as the hypoxia-response element (HRE) and transactivates a variety of hypoxia-responsive genes.^9^ As the transcriptional activator of erythropoietin, HIF-2 controls intracellular hypoxic responses throughout the body.

HIF-2*α* is expressed in endothelial cells, the parenchyma and interstitial cells of multiple organs.^1, 10, 11^ Although HIF-2*α* is stabilized at higher O_2_ pressure compared with HIF-1*α in vitro*, it was not detected under normoxic conditions in various organs.^11, 12, 13^ However, HIF-2*α* was shown to be markedly induced under hypoxia in all organs investigated, including brain, heart, lung, kidney, liver, pancreas and intestine.^11^

Hypoxia is a common phenomenon in many tumors. The HIF-1*α* and HIF-2α proteins are expressed in most types of human tumors, including breast, colon, ovarian, pancreatic, prostate, renal and hepatocellular cancers.^14, 15, 16^ Compared with surrounding normal tissues, HIF-1*α* or HIF-2*α* (or both) have been detected at higher levels in the majority of primary human cancers and their metastases.^14, 15, 16, 17^ HIF-2*α* mediates the adaptive response to decreased O_2_ availability at the cellular and organismal level.

## HIF-2 Is Different from HIF-1

The HIF transcription factors mediate the primary transcriptional responses to hypoxic stress in normal and transformed cells. Both HIF-1*α* and HIF-2*α* are O_2_-labile *α*-subunits and heterodimeric complexes composed of bHLH-PAS proteins. In addition, both of them can bind ARNT and mediate transcription. However, increasing evidence has indicated that HIF-2 is distinct or even opposite in many ways from HIF-1.^12, 18, 19, 20^ To ease understanding, we here delineate the differences between HIF-2 and HIF-1 in three parts. In fact, all the disparities are cause-and-effect relationships and should be conceived as part of a whole in the cellular processes.

First, HIF-2*α* expression has been shown to differ from that of HIF-1*α* (Table 1) in various tissues. In contrast to HIF-1*α* that appears to be expressed in nearly all cell types, HIF-2*α* expression is restricted to specific types, including endothelial cells, glial cells, type II pneumocytes, cardiomyocytes, kidney fibroblasts, interstitial cells of the pancreas and duodenum and hepatocytes. In the umbilical cord, HIF1*α* is expressed in smooth muscle cells that surround blood vessels, whereas HIF-2*α* was detected in endothelial cells that line blood vessel walls.^1, 2, 3^ HIF-1*α* was observed by cytoplasmic staining in addition to nuclear accumulation; HIF-2*α* is confined to the cell nucleus and expressed only under hypoxic stimulation. Although HIF-1*α* is expressed in renal tubules and neuronal cells, HIF-2*α* was detected in nonparenchymal cells including glomerular, kidney peritubular endothelial cells and fibroblasts, and brain endothelial and glial cells.^11^ Furthermore, HIF-2*α* expression has been shown to differ from that of HIF-1*α* in many cancers. In addition, HIF-2 is expressed in tumor-associated macrophages, although HIF-1*α* distribution tends to be more restricted to perinecrotic regions in renal cell carcinoma (RCC).^14^

Another difference concerns the oxygen conditions needed for expression. HIF-1*α* was shown by immunohistochemistry to be already expressed under normoxic baseline conditions, unlike HIF-2*α.*^11^ More severe hypoxia (6% O_2_) is required to induce HIF-1*α* compared with HIF-2*α* in liver and kidney.^11^ HIF-1*α* is most active during short periods (2–24 h) of intense hypoxia or anoxia (<0.1% O_2_), whereas HIF-2*α* was shown to be active under mild or physiological hypoxia (<5% O_2_) in both SK-N-BE(2)c and KCN-69n neuroblastoma cell lines.^12^ These findings indicate that HIF-2 plays a critical role in driving the hypoxic response, whereas HIF-1 controls the initial response to hypoxia in certain contexts.^12, 20^

The two proteins also differ by the time needed for activation and duration of activity. HIF-1*α* induction in kidney and liver was shown to be transient; however, HIF-2*α* expression is sustained.^11^ Moreover, HIF-1*α* was transiently stabilized and primarily mediated acute responses, whereas HIF-2*α* gradually accumulated and managed prolonged hypoxic gene activation under hypoxia (1% O_2_) in neuroblastoma. The activity of HIF-2*α* persists even after 48–72 h of hypoxia in neuroblastoma cell lines.^12^

Second, based on their distinct expression patterns, HIF-2*α* plays different roles in many respects compared with HIF-1*α* (Table 2). It was found that the majority of hypoxia-induced genes contained HIF-1-binding sites, and gene expression was shown to be dependent on HIF-1*α* expression. HIF-2*α* binding was redundant for many genes, but knockdown of HIF-2*α* levels did not affect gene expression.^21^ In fact, emerging studies have shown that HIF-1*α* and HIF-2*α* regulate different target genes, with non-redundant and even opposite biological functions. HIF-1*α* plays more important roles in endothelial cell proliferation, migration and vessel sprouting; HIF-2*α* plays a more significant role in controlling vascular morphogenesis, integrity and assembly.^22^ Overexpression of HIF-2*α* was shown to enhance the expression of endothelial tyrosine kinase receptor *Tie2*, in contrast to HIF-1*α.*^1^ HIF-2*α* or other hypoxia-induced factors cannot compensate for the loss of HIF-1*α*, whereas hypoxic induction of HIF-1*α* target genes is attenuated in Hif-1*α*-deficient endothelial cells.^23^ Interestingly, HIF-1*α* decreased IL-8 expression whereas HIF-2*α* overexpression increased the mRNA and protein levels of this cytokine in HMEC-1 cells.^24^ HIF-2*α* also seems to have a stronger transactivation activity than HIF-1*α* on vascular endothelial growth factor (VEGF) promoter.^2, 13, 25, 26^ Moreover, HIF-2 is the dominant HIF regulating VEGF and other angiogenic factors in mouse liver hemangiomas.^27^ HIF-1*α* facilitates cell growth *in vitro* and tumorigenesis *in vivo*, whereas HIF-2 showed opposite effects in colon cancer cells.^28^ Surprisingly, HIF-2*α* also has distinct or even opposite functions with HIF-1*α* in some cancers. In RCC, HIF-2*α*, but not HIF-1*α*, promotes tumor growth in xenograft models. Overexpression of stable HIF-1*α* inhibits tumor growth in 786-O RCC cells,^29, 30^ whereas overexpression of stable HIF-2*α*-expressing pVHL restores xenograft growth to the level of parental VHL-null cells.^30, 31, 32^ HIF-2*α* expression was shown to increase with the degree of dysplasia in preneoplastic kidney lesions of patients with VHL disease, whereas HIF-1*α* expression decreased, indicating that HIF-2*α* plays a role in the transformation of dysplastic cells.^30, 33^ A similar phenomenon was also observed in hepatocellular carcinoma (HCC) and colorectal cancers as described, though the data did not allow firm conclusion. HIF-1*α* deficiency inhibited overall tumor growth, whereas deficiency of HIF-2*α* stimulated tumor growth in colorectal cancers xenograft.^28^ HIF-1*α* and HIF-2*α* are differentially regulated *in vivo* and reflected distinctive protein expression patterns and stabilization mechanisms in HCC.^15, 34^ HIF-2*α* also appears to have a more general role in promoting tumorigenesis. Subcutaneous teratomas generated from ES cells with the HIF-2*α* cDNA ‘knocked in' into the Hif-1*α* locus exhibited a fourfold mass increase compared with WT RCC 786-O cells controls, mainly because of increased proliferation.^35^ HIF-2*α* was shown to be stable under prolonged and mild hypoxia conditions in neuroblastoma, and may promote angiogenesis even in tumors experiencing minimal hypoxic stress.^12^ Loss of HIF-1*α* in endothelial cells reduces NO synthesis, retards tumor cell migration through endothelial layers and restricts tumor cell metastasis; however, loss of HIF-2*α* resulted in opposite effect in each case.^36^ Deficiency of HIF-2*α* increased tumor growth and progression, whereas HIF-1*α* deletion had nearly no effect on tumor burden and progression in a KRAS-driven lung tumor model.^37^ HIF-2*α* signaling, but not HIF-1*α*, may contribute to tumor angiogenesis via autocrine transforming growth factor-*β*1 (TGF-*β*1) production under nonhypoxic conditions in prostate tumor cells.^38^ For cancer stem cells (CSCs), though hypoxia and HIFs may contribute to the maintenance of putative cancer ‘stem' cells, HIF-2*α* seemed also to play a distinct role from HIF-1*α*, through unique mechanisms. A recent study revealed that HIF-2*α* regulates Oct4 directly upstream and binds hypoxic regulatory elements in the promoter of murine Oct4, which is a stem cell regulatory protein; this is not the case for HIF-1*α.*^39^ HIF-2*α* may have a specific function in glioblastoma stem cells: it was shown to be selectively expressed in the CD133+ subpopulation of glioblastoma cells, whereas HIF-1*α* expression is widespread among both tumorigenic and nontumorigenic cells.^40^

**Figure 2:**
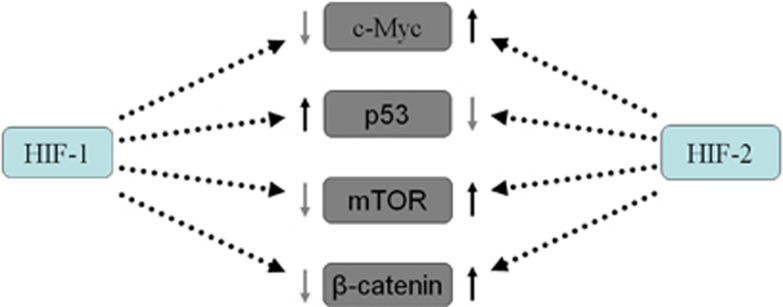
HIF-1 and HIF-2 regulate conversely some key downstream genes. HIF-2 increases *c-Myc*, mTOR and *β*-catenin activity and decrease p53 activity, whereas HIF-1 exert opposite effects

**Figure 3:**
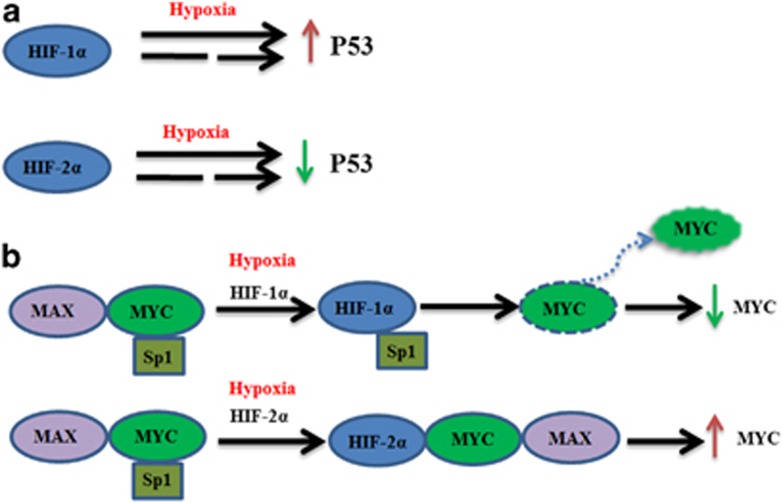
(**a**) HIF-1*α* upregulates P53 activity whereas HIF-2*α* downregulates P53 activity under hypoxia directly (full line) or indirectly (dotted line). (**b**) HIF-1*α* and HIF-2*α* regulate distinctively c-Myc interacting with its transcription cofactors. HIF-1*α* diminishes the association with MAX and SP1, and then decreases MYC activity, whereas HIF-2*α* combines with MAX and SP1 complex, thereby increasing MYC activity

Third, HIF-2*α* and HIF-1*α* regulate key downstream genes, including *c-Myc*, *p53*, *mTOR* and *β*-catenin, conversely (Figure 2). For example, HIF-1*α* and HIF-2*α* showed opposite effects on c-Myc interaction with its transcription cofactors. HIF-1*α* induction was demonstrated to inhibit c-Myc transcriptional activity and suppress cell proliferation.^24, 41, 42^ However, HIF-2*α* induction promotes cell cycle progression by enhancing c-Myc function.^24, 43^ In addition, HIF-2*α*-mediated suppression of p53 is central to maintenance of the enhanced stemness phenotype in human embryonic stem cells. Unlike HIF-2*α*, HIF-1*α* expression declined markedly following reoxygenation in the model^44, 45^(Figure 3). HIF-2*α* may regulate the focal adhesion kinase family interacting protein of 200 kD gene and stimulate mTORC1 to promote cellular proliferation in O_2_-deprived cells.^46^ On the contrary, HIF-1*α* induces DDIT4 gene expression and represses mTORC1 by promoting the release of sequestered TSC2 from 14-3-3 proteins.^47, 48^ A recent study found that HIF-1 negatively regulates Wnt/*β*-catenin signaling by sequestering *β*-catenin from *β*-catenin/T-cell factor (TCF); instead, HIF-2α interacts with *β*-catenin at a different site and assembles with *β*-catenin/TCF to facilitate gene transcription.^49^

## HIF-2*α* in Digestive System Cancers

**Figure 4:**
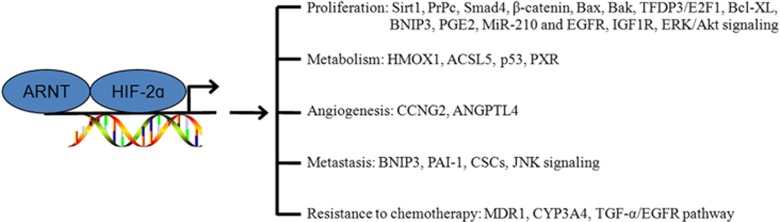
Mechanisms underlying HIF-2*α* effect on tumorigenesis in digestive system cancer. HIF-2*α* regulates genes that modulate key aspects of tumorigenesis, including proliferation, metabolism, angiogenesis, metastasis and resistance to chemotherapy

HIF-2*α* is frequently detected in most types of solid tumors, including head and neck, renal, bladder, glial, breast, ovarian, prostate and renal cancers.^14, 15, 25, 28, 50, 51, 52, 53^ An increasing number of studies reporting HIF-2 in the context of digestive system cancer have emerged in recent years and conveyed that this protein plays important roles in digestive system cancers. Interestingly, its prognostic roles are explicit in pancreatic and gastric cancers but confused in colon and hepatocellular cancers (Table 3). HIF-2 upregulates several genes involved in a variety of tumorigenesis events: cell proliferation, angiogenesis, metabolism, metastasis and resistance to chemotherapy (Figure 4).

## Proliferation

Studies have suggested that HIF-2 can regulate cell proliferation through multiple mechanisms, although the outcomes are not identical for all cancers and even controversial in some cases. HIF-2 was shown to be necessary and sufficient to maintain tumor growth in VHL-deficient RCC cells.^31, 32^ HIF-2 was shown to control cellular proliferation through modulation of c-Myc activity and cyclin D1 in RCC cells.^30, 54^ In addition, new mechanisms for HIF-2 were described in digestive system cancer.

A recent study showed that HIF-2 plays opposite roles in different digestive tract cancer cell lines through Sirt1. Sirt1 inhibited HCT116 cell growth but promoted HepG2 cell growth. The cell type-dependent actions of Sirt1 on cell growth may be attributable to its opposite effects on HIF-2*α* activity in different cell lines.^18^ PrPc (cellular prion protein) is a glycosylphosphatidylinositol anchored membrane protein with various physical functions. PrPc is highly expressed in colorectal adenocarcinomas. It is involved in regulating Glut1 expression through the Fyn-HIF-2α pathway.^55^

By maintaining appropriate levels of both Smad4 and *β*-catenin, HIF-2*α* modulates the Wnt signaling pathway during mPanIN progression, with the oncogenic protein Ras expressed in the pancreas. Loss of Hif-2*α* in mice instead led to markedly higher number of mPanIN lesions.^56^ The average amounts of HIF-2*α* at both protein and mRNA levels were significantly lower in tumor than peritumoral tissues in HCC, and the decreased levels were associated with lower overall survival.^57^ Bax and Bak were expressed at higher levels in HCC SMMC-7721 cells with high HIF-2*α* expression relative to controls.^57^ Both Bax and Bak are pro-apoptotic BCL-2 proteins and would facilitate the release of cytochrome *c*. The latter triggers cleaved caspase 3, which is also a key executer of apoptosis detected at higher levels in cells overexpressing HIF-2*α.*^58, 59^ These data confirmed that high levels of HIF-2*α* in HCC cells cause cell growth arrest through apoptosis. Furthermore, it was suggested that HIF-2*α* induces apoptosis through a novel TFDP3/E2F1 pathway involving both p53-dependent and -independent modes. Hence, high HIF-2*α* expression in HCC was shown to be correlated with a good outcome.^57^. Menrad *et al.*^60^ found that knockdown of HIF-1*α* or HIF-2*α* increased cell viability as well as spheroid size and decreased caspase-3 activity. Indeed, HIF-2*α*-knockdown cells upregulated HIF-1*α* and enhanced Bcl-XL and BNIP3 expression. BNIP3 binds to the Beclin-1/Bcl-XL complex, thereby releasing Beclin-1 that subsequently induces autophagosome formation.^61, 62^ However, the role of HIF-2*α* in the development of cancer is unclear, and inconsistencies abound in the existing literature. Bangoura *et al.*^15^ reported that HIF-2*α* is overexpressed in pericarcinoma tissues in HCC and significantly correlated with tumor grade and reduced survival in patients with HCC. HIF-2*α*/EPAS1 itself, to some extent, affects patient survival.^15^

HIF-2*α* is overexpressed in colon cancer,^14, 17^ and its activation *in vivo* directly upregulates COX2 expression and facilitates colon tumorigenesis. HIF-2*α* activates COX2/mPGES-1/PGE2 signaling to facilitate colon tumorigenesis.^63^ In addition, HIF-2*α* exerts its proliferative effects through modulating the EGFR, IGF1R and ERK/Akt signaling pathways in colorectal carcinoma, and its inhibition prevents growth and tumorigenesis of colorectal cancer *in vivo.*^64^ However, an opposite outcome was reported, with HIF-2*α*-selective knockdown having no effect on cellular proliferation in colorectal cells, although colony formation doubled in soft agar assays.^28^

A research has also investigated the relationship between HIF-2*α* and microRNA (miRNA) in digestive system cancers. HIF-2*α* was shown to mediate miR-210 and *c-Myc* to participate in neoplasma. MicroRNA-210 is a direct transcriptional target of HIF-2*α* and its upregulation led to a switch from Mnt to c-Myc expression during cholestatic cholangiocarcinogenesis *in vivo.*^65^

At present, inconsistent conclusions have been reached for the role of HIF-2 in digestive system cancers, especially in HCC and colorectal carcinoma. The function of HIF-2*α* appears to be cell-type dependent. The different expression patterns and levels of HIF-2 as well as cell-specific cofactors may affect its activity. Indeed, the organism environment is very complex and HIF-2 could be influenced by many other factors. For instance, HCC is often complicated with hepatitis B or C and hepatocirrhosis that may also promote a hypoxic response that stabilizes HIFs in oxygen tension.^66, 67^ Therefore, HIF-2 may also be involved in these diseases, leading to a complicated network in certain organs.

## Metabolism

HIF-2*α* plays a critical role in metabolism, modulating the expression of cytochrome *c* oxidase isoforms so as to maximize the efficiency of the electron transport chain. Defects in this response lead to impaired ATP production and elevated oxidant production in hypoxia.^68^ HIF-2*α*/ARNT targets (e.g., SOD2) also protect cellular and mitochondrial components during oxidative stress in mice.^69^

Cancer cells shift glucose metabolism from oxidative route to glycolytic pathway, which involves decreased mitochondrial respiration and increased lactate production, even in the presence of oxygen.^70, 71^ HIF-*α* target genes are involved in the regulation of numerous pathways important for tumor metabolism.^72^

HIF-2*α* depletion downregulated the expression of heme oxygenase (*HMOX1*) genes that exert antioxidant functions in RCC.^73^ In colon cancer cells, absence of oncogenic KRAS or both HIF-1*α* and HIF-2*α* led to decreased cardiolipin level and inefficient mitochondrial respiration; ACSL5 induction is directly responsible for maintaining cardiolipin level and efficient mitochondrial respiration in cancer. Colon cancer cells with oncogenic *KRAS* mutation and expressing both HIF-1*α* and HIF-2*α* were shown to maximize ATP production and minimize ROS generation, probably through the induction of enzymes important for mitochondrial cardiolipin synthesis.^74^

Poorer survival of HIF-2*α* and wild-type *TP53* was associated with carbonic anhydrase 9 (CA9) stromal-positive colorectal adenocarcinomas. Furthermore, tumors expressing HIF-2*α* or CA9 in their stroma show poorer prognosis in wild-type *TP53* tumors compared with mutant malignancies. It is plausible that *p53* is involved in the metabolic switch to glycolysis, when oxidative phosphorylation is impaired during hypoxia;^75^ *p53* may simply correlate with defects in another pathway such as the BNIP3 cell death pathway that substitutes for *TP53* loss in a similar manner during carcinogenesis.^76^ CYP (cytochrome *P*450) 3A4 mediates exogenous-drug transportation and oxidative metabolism. We have observed that HIF-2*α* modulates the recruitment of pregnane X receptor (PXR) to the PXR response element in the *CYP3A4* (cytochrome *P*450 3A4) gene promoter region in gastric cancer BGC-823 cells (Jiuda Zhao, unpublished data).

## Angiogenesis

Angiogenesis plays an important role in tumorigenesis and cancer progression. Hypoxia is the prime driving force for tumor angiogenesis, and HIFs play a key role in this process. Growing evidence supports that HIF-2*α* is involved in angiogenesis.^77^ HIF2 expression was directly correlated with microvessel density and cyclooxygenase 2 expression in colorectal carcinoma, indicating its potential role in angiogenesis of colorectal carcinomas.^53^ Selective knockdown of HIF-2*α* resulted in more pronounced decrease of VEGF and MVD levels compared with HIF-1*α* repression in colon cancer xenograft studies. HIF-2*α* exerts these unique effects on colonic tumorigenesis through cyclin G2 (CCNG2) and angiopoietin-like 4 (ANGPTL4), a secreted protein of the angiopoietin-like family induced by hypoxia.^28^ HIF-2 upregulates the expression of VEGF that binds VEGFR and stimulates tumor angiogenesisin in HCC.^77^ Transfection of HIF-2*α* siRNA into HCC cells was shown to downregulate the expression of VEGF, cyclin D1, HIF-2*α* and TGF-*α*, inhibiting the activation of EGFR.^78^ Conditional inactivation of HIF-2*α* resulted in suppressed development of VHL-associated liver hemangiomas; in addition, angiogenic gene expression in hepatocytes is predominantly regulated by HIF-2 and not HIF-1. It suggests that HIF-2 is the dominant HIF in the pathogenesis of VHL-associated vascular tumors. Hemangioma formation does not require HIF-1*α*, but is dependent on ARNT, suggesting that HIF-2*α* may play an essential role in VHL-associated vascular tumorigenesis.^79^ These observations indicate that HIF-2*α* is an important mediator of angiogenesis in digestive tract cancers.

## Metastasis

Hypoxia is an important microenvironmental factor that induces cancer metastasis. HIF activation correlates with metastasis in multiple tumors and can promote metastasis through the regulation of key factors governing tumor cell metastatic potential, including E-cadherin, *LOX*, *CXCR4*, *SDF-1* and *TWIST* in RCC, breast and head/neck cancers.^80, 81, 82, 83, 84, 85^

Some studies have indicated that HIF-2*α* also participates in digestive tract cancer metastasis by regulating the JNK signaling pathway, BNIP3, *PAI-1* and CSCs. In gastric cancer, HIF-1*α* and HIF-2*α* levels are higher in metastasis samples compared with non-metastatic ones. Hypoxia (1% O_2_, 8 h) was shown to induce HIF-1*α* and HIF-2*α* expression in gastric cancer cell lines. Small interfering RNA (siRNA) against HIF-1*α* and HIF-2*α* in gastric cancer cells significantly inhibited hypoxia-induced adhesive and invasive abilities. HIF-2*α* is therefore involved in metastasis and invasion of gastric cancer cells under hypoxia, in a mechanism involving the JNK signaling pathway.^86^ In primary colorectal cancer, overexpression of HIF-2*α* together with BNIP3 was linked to local invasion and lymph node metastasis.^87^

The role of HIF-2*α* in HCC is not entirely understood. Some studies have demonstrated that HIF-2*α* is associated with tumor metastasis, whereas others hold the opposite opinion. A study found that HIF-2*α* is overexpressed in HCC compared with noncancerous lesions, and its levels were significantly correlated with tumor grade, venous invasion, intrahepatic metastasis, necrosis and capsule infiltration.^15^ Two HREs at −3.6 kb of the 5'-flanking promoter region of the *PAI-1* gene can function as *cis*-acting elements to regulate *PAI-1* gene induction by hypoxia in mouse hepatoma cells. The interaction of these HREs with the HIF-1 or HIF-2 protein mediates the transcription of the *PAI-1* gene that is involved in invasion and metastasis.^88^ However, Yang *et al.* reported recently that high HIF-2α expression was found in only 13.5% (17/126) of tumors compared with 47.6% (60/126) of peritumoral tissues in HCC samples. There was no relationship between HIF-2*α* and capsular infiltration or portal vein invasion in HCC patients.^34^

Another mechanism by which HIF-2 controls metastasis is through modulation of CSCs. It is widely accepted that metastasis originates from CSCs with tumor-initiating capabilities that allow most disseminated tumor cells to reconstitute growing, heterogeneous secondary tumors.^89^ Blocking HIF-1*α* or HIF-2*α* activity results in dramatically decreased CSC proliferation and self-renewal in hematological malignancies and glioma stem cells.^40, 90, 91, 92^ Limited research has indicated that HIF-2*α* also plays roles in metastasis of digestive tract cancers by regulating the CSCs. Interestingly, HIF-2*α* co-stained with CD133 in pancreatic ductal adenocarcinoma (PDA), suggesting that tumor hypoxia is associated with the expression of CSC markers in PDA. Hypoxia-driven EMT occurs in tumor tissues from pancreatic cancer patients, with CSC-positive tumor cells present in hypoxic tumor microenvironments. Only stem-like cells acquire high migratory potential and thus may be responsible for invasion and metastasis.^93^

## HIF-2 and Resistance to Chemotherapy

The hypoxic environment plays a critical role in promoting resistance to anticancer drugs.^94, 95^ Previous studies have indicated that HIF-2 responds rapidly to decreased oxygen levels to activate the expression of a broad range of genes that promote resistance.

In the case of colon cancer cells, oxaliplatin increased HIF-2*α* accumulation and enhanced cell growth; indeed, HIF-2*α* may mediate, at least partly, oxaliplatin resistance in SW1116 cells.^96^ Disruption of HIF-1*α* or HIF-2*α* gene, or both, further improved the tumor response to sunitinib therapy in human colon cancer cells.^97^ Upregulation of HIF-2*α* induced by sorafenib was shown to contribute to resistance of hypoxic HCC cells by activating the TGF-*α*/EGFR pathway.^79^

To better understand the roles of HIF-2 in chemotherapy resistance, our laboratory studied the relationship between HIF-2*α* and multiple drug resistance (MDR1). We found that overexpression of HIF-2*α* inhibits the PXR transcriptional activity and reduces the expression of PXR downstream genes, whereas increasing hypoxia elicited MDR1 and CYP3A4 expression. Moreover, overexpression of HIF-2*α* significantly reduced the pharmacological effects of Paclitaxel, Mitomycin C, Imatinib and Sorafenib on gastric cancer BGC823 cells, and the corresponding IC_50_ values increased markedly (Jiuda Zhao, unpublished data). Therefore, HIF-2 may serve as a biomarker for a better understanding of chemoresistance in digestive system cancer through *MDR1* gene induction.

Taken together, these experimental and clinical data delineate an important role for HIF-2 in chemotherapy resistance through multiple mechanisms and provide a basis for reversing drug resistance with HIF-2 inhibitors.

## HIF-2 and Tumor Inhibition

As HIF complexes are involved in cancer cell adaptation to hypoxic tumor microenvironments, inhibitors of the HIF signaling pathway have been developed and used to inhibit the expression of HIF target genes and reduce angiogenesis and metastasis.^98^ The ability to selectively inhibit HIF activity would be beneficial to clinical treatment.^99^ A large collection of compounds have been reported to inhibit HIF transcriptional activity, either directly or indirectly.^100, 101, 102^ For example, downregulation of the oncoprotein human double minute 2 reduced the levels of HIF-1 and HIF-2*α* in a *p53*- and VHL-independent manner.^103^ The JNK inhibitor SP 600125 abolished hypoxia-induced HIF-1*α* and HIF-2*α* expression, and inhibited the adhesive and invasive abilities of gastric cancer cells.^86^ Two new cembrane diterpenes were found to selectively inhibit HIF-2*α* and modulate downstream effectors of tumorigenesis.^104^

In addition, agents that inhibit reactive oxygen species generation, such as superoxide dismutase mimetics, have also been shown to decrease HIF levels.^105^ Inducing PHD enzyme activity with derivatives of *α*-ketoglutarate is also a method to target hypoxic areas of tumors, although it reduces signaling through both HIF-1*α* and -2*α.*^46^ Overexpression of the tumor suppressor protein pVHL inhibits HCC growth in mice by downregulating HIF-1*α* and HIF-2*α.*^106^

To date, only few inhibitors have been shown to selectively target HIF-2*α*. Furthermore, although laboratory effects of chemical and biologic inhibitors of HIF-2α on tumor cells are being investigated, clinical grade HIF-2*α*-specific inhibitors have yet to be developed.

## Conclusion and Perspectives

In conclusion, multitudinous studies have provided persuasive evidence that HIF-2 plays important roles in many critical aspects of digestive system cancers. It is involved in cellular proliferation, metabolism, angiogenesis, metastasis and resistance to drugs. Clinical data also indicated that HIF-2 overexpression is associated with prognosis of digestive system cancers. The HIF-2 pathway may therefore constitute a useful biomarker for assessing disease states as well as developing cancer treatments.

However, multiple questions regarding HIF-2 need to be promptly answered. First, inconsistent associations between HIF-2 and digestive tract cancers have been reported. The study conclusions do not corroborate each other, sometimes for the same organ. This suggests that HIF-2-specific gene activating functions respond differently to various stimuli, with gene- or tissue-specific regulatory mechanisms involving additional transcription factors. Moreover, the limited sample size in different studies and different cell line status in some studies may also affect the results. Second, although data have been amassed delineating the mechanisms and consequences of increased HIF-2 activity during cancer progression, its definite mechanisms remain elusive in digestive system cancers. The role of HIF-2*α* in the pathogenesis of cancers is extensively studied in RCC, but far too few studies have assessed HIF-2*α* in digestive system cancers. Third, the exploitation of HIF-2*α* inhibitors is still challenging. Most inhibitors target nonselectively both HIF-1*α* and HIF-2*α*. HIF targeting involves the overlapping but distinct biological roles of the HIF-*α* subunits. Compounds that promote the binding of IRP1 to the 5′ UTR of HIF-2*α* mRNA decrease HIF-2*α* hypoxic induction, but also repress HIF-1*α* synthesis via an independent mechanism.^107^ Discriminating between the *α-*subunits and their relative contributions to different digestive system cancers will be important for appropriately testing compounds for use in therapy. The intricate interplay between HIF-1 and HIF-2 should also be studied further. In addition, although another HIF family member, HIF-3, can also dimerize with ARNT and bind to HREs *in vitro*,^108^ its role in the hypoxic regulation of target gene expression and interaction with HIF-1 and HIF-2 *in vivo* is not well understood. We believe that further studies are warranted to elucidate the important role of HIF-2 in digestive system cancers. There is still a long way to go before one can confidently promote the clinical application of HIF-2 targeting therapy for digestive system cancers.

## Figures and Tables

**Table 1:** Different expression of HIF-1*α* and HIF-2*α*

**HIF-1*****α***	**HIF-2*****α***	**References**
*Tissue distribution*		
Nearly all cell types	Endothelial cells, glial cells, type II pneumocytes, cardiomyocytes, fibroblasts of the kidney, interstitial cells of the pancreas and duodenum and hepatocytes	^1, 2, 3^
Umbilical cord smooth muscle cells	Endothelial cells	^1, 2, 3^
Cytoplasm and cell nuclei	Cell nuclei	^1, 2, 3^
Renal tubules and neuronal cells	Kidney glomerular, peritubular endothelial cells and fibroblasts, brain endothelial cells and glia cells	^11^
No	Neuroblastoma well-vascularized areas	^12^
RCC perinecrotic regions	RCC tumor-associated macrophages	^14^
		
*Oxygen condition*		
Normoxic baseline conditions	No	^11^
More severe hypoxia (6% O_2_) in liver and kidney	No	^12^
		
*Time to be activated and duration*		
Transient in kidney and liver	Sustained in kidney and liver	^11, 12^

**Table 2:** Different functions and related target genes between HIF-1 and HIF-2

**HIF-1*****α***	**HIF-2*****α***	**Function**	**Gene**	**Cell type**	**References**
+	−	Endothelial cell proliferation, migration, and vessel sprouting	*CXCR4*	Endothelial cell	^22^
−	+	Controlling vascular morphogenesis, integrity, and assembly	*CXCR4*	Endothelial cell	^22^
−	+	Regulates embryonic vasculature	*Tie2*	Embryonic kidney cells	^1^
−	+	Increases IL-8 expression	*c-Myc, Mxi-1*	HMEC-1 cells	^24^
−	+	A stronger transactivation activity on VEGF promoter	*(HIF-2 DNA binding sequences)*	Hepa-1,Hep3, Ka13, fetal kidney cell line mouse,MG63 Vhlh-deficient mice	^2, 13, 25^
+	−	Facilitates cell growth and tumorigenesis	*CCNG2,ANGPTL4*	SW480, DLD-1 cells	^28^
−	+	Promotes tumor growth	*BNip3 transcriptional regulator*	RCC	^29, 30, 31, 32^
−	+	Transformation of dysplastic cells	*Cyclin D1, GLUT-1,*	RCC	^30, 33^
−	+	Increases proliferation	*VEGF, TGF-α*	Endothelial cells	^35^
−	+	Retards tumor cell migration	*iNOS*	Endothelial cells	^36^
−	+	Angiogenesis	*TGF-β1*	Prostate tumor	^38^
−	+	Regulates stem cell	*Oct4, CD133*	Embryos, glioblastoma	^39, 40^

**Table 3:** HIF-2 activation in digestive system cancer

**Cancer**	**HIF-2*****α***	**Prognosis**		**References**
		**Poor**	**Good**	
Gastric	↑	+	−	^18, 86^
Hepatocellular	↑	+	−	^14, 15, 34, 78^
	↑	−	+	^60^
	↓	+	−	^57^
Pancreatic	↑	+	−	^14, 56^
Colorectal	↑	+	−	^14, 52, 64, 76, 87^
	↓	+	−	^28^

The symbol ↑ indicates upregulation, ↓ indicates downregulation; + indicates there is correlation and − indicates there is no correlation

## Abbreviations

CA9: carbonic anhydrase 9
CSC: cancer stem cell
CYP3A4: cytochrome *P*450 3A4
EPAS1: endothelial PAS domain protein-1
HCC: hepatocellular carcinoma
HIF: hypoxia-inducible factor
HRE: hypoxia-response element
MDR1: multiple drug resistance
PrPc: cellular prion protein
PDA: pancreatic ductal adenocarcinoma
PHD: prolyl hydroxylase-domain enzyme
pVHL: von Hippel–Lindau protein
PXR: pregnane X receptor
RCC: renal cell carcinoma
VEGF: vascular endothelial growth factor

## References

[bib1] Tian H, McKnight SL, Russell DW. Endothelial PAS domain protein 1 (EPAS1), a transcription factor selectively expressed in endothelial cells. Genes Dev 1997; 11: 72–82. 9000051 10.1101/gad.11.1.72

[bib2] Ema M, Taya S, Yokotani N, Sogawa K, Matsuda Y, Fujii-Kuriyama Y. A novel bHLH-PAS factor with close sequence similarity to hypoxia-inducible factor 1alpha regulates the VEGF expression and is potentially involved in lung and vascular development. Proc Natl Acad Sci USA 1997; 94: 4273–4278. 9113979 10.1073/pnas.94.9.4273 PMC20712

[bib3] Flamme I, Fröhlich T, von Reutern M, Kappel A, Damert A, Risau W. HRF a putative basic helixloop-helix-PAS-domain transcription factor is closely related to hypoxia-inducible factor-1 alpha and developmentally expressed in blood vessels. Mech Dev 1997; 63: 51–60. 9178256 10.1016/s0925-4773(97)00674-6

[bib4] Hogenesch JB, Chan WK, Jackiw VH, Brown RC, Gu YZ, Pray-Grant M et al. Characterization of a subset of the basic-helix-loop-helix-PAS superfamily that interacts with components of the dioxin signaling pathway. J Biol Chem 1997; 272: 8581–8593. 9079689 10.1074/jbc.272.13.8581

[bib5] Gordan JD, Simon MC. Hypoxia-inducible factors: central regulators of the tumor phenotype. Curr Opin Genet Dev 2007; 17: 71–77. 17208433 10.1016/j.gde.2006.12.006 PMC3215290

[bib6] Mahon PC, Semenza GL. FIH-1: a novel protein that interacts with HIF-1alpha and VHL to mediaterepression of HIF-1 transcriptional activity. Genes Dev 2001; 15: 2675–2686. 11641274 10.1101/gad.924501 PMC312814

[bib7] Jaakkola P, Mole DR, Tian YM, Wilson MI, Gielbert J, Gaskell SJ et al. Targeting of HIF-alpha to the von Hippel-Lindau ubiquitylation complex by O2-regulated prolyl hydroxylation. Science 2001; 292: 468–472. 11292861 10.1126/science.1059796

[bib8] Ohh M, Park CW, Ivan M, Hoffman MA, Kim TY, Huang LE et al. Ubiquitination of hypoxia-inducible factor requires direct binding to the beta-domain of the von Hippel-Lindau protein. Nat Cell Biol 2000; 2: 423–427. 10878807 10.1038/35017054

[bib9] Maxwell PH, Pugh CW, Ratcliffe PJ. Activation of the HIF pathway in cancer. Curr Opin Genet Dev 2001; 11: 293–299. 11377966 10.1016/s0959-437x(00)00193-3

[bib10] Tian H, Hammer RE, Matsumoto AM, Russell DW, McKnight SL. The hypoxia-responsive transcription factor EPAS1 is essential for catecholamine homeostasis and protection against heart failure during embryonic development. Genes Dev 1998; 12: 3320–3324. 9808618 10.1101/gad.12.21.3320 PMC317225

[bib11] Wiesener MS, Jurgensen JS, Rosenberger C, Scholze CK, Horstrup JH, Warnecke C et al. Widespread hypoxia-inducible expression of HIF-2alpha in distinct cell populations of different organs. FASEB J 2003; 17: 271–273. 12490539 10.1096/fj.02-0445fje

[bib12] Holmquist-Mengelbier L, Fredlund E, Lofstedt T, Noguera R, Navarro S, Nilsson H et al. Recruitment of HIF-1alpha and HIF-2alpha to common target genes is differentially regulated in neuroblastoma: HIF-2alpha promotes an aggressive phenotype. Cancer Cell 2006; 10: 413–423. 17097563 10.1016/j.ccr.2006.08.026

[bib13] Wiesener MS, Turley H, Allen WE, Willam C, Eckardt KU, Talks KL et al. Induction of endothelial PAS domain protein-1 by hypoxia: characterization and comparison with hypoxia-inducible factor-1alpha. Blood 1998; 92: 2260–2268. 9746763

[bib14] Talks KL, Turley H, Gatter KC, Maxwell PH, Pugh CW, Ratcliffe PJ. The expression and distribution of the hypoxia-inducible factors HIF-1a and HIF-2a in normal human tissues, cancers, and tumor-associated macrophages. Am J Pathol 2000; 157: 411–421. 10934146 10.1016/s0002-9440(10)64554-3 PMC1850121

[bib15] Bangoura G, Liu ZS, Qian Q, Jiang CQ, Yang GF, Jing S. Prognostic significance of HIF-2alpha/EPAS1 expression in hepatocellular carcinoma. World J Gastroenterol 2007; 13: 3176–3182. 17589895 10.3748/wjg.v13.i23.3176 PMC4436602

[bib16] Shi CY, Fan Y, Liu B, Lou WH. HIF1 contributes to hypoxia-induced pancreatic cancer cells invasion via promoting QSOX1 expression. Cell Physiol Biochem 2013; 32: 561–568. 24008827 10.1159/000354460

[bib17] Zhong H, De Marzo AM, Laughner E, Lim M, Hilton DA, Zagzag D et al. Overexpression of hypoxia-inducible factor 1alpha in common human cancers and their metastases. Cancer Res 1999; 59: 5830–5835.]. 10582706

[bib18] Yoon H, Shin SH, Shin DH, Chun YS, Park JW. Differential roles of Sirt1 in HIF-1α and HIF-2α mediated hypoxic responses. Biochem Biophys Res Commun 2014; 444: 36–43. 24423936 10.1016/j.bbrc.2014.01.001

[bib19] Eubank TD, Roda JM, Liu H, O'Neil T, Marsh CB. Opposing roles for HIF-1α and HIF-2*α* in the regulation of angiogenesis by mononuclear phagocytes. Blood 2011; 117: 323–332. 20952691 10.1182/blood-2010-01-261792 PMC3037754

[bib20] Koh MY, Lemos R Jr, Liu X, Powis G. The hypoxia-associated factor switches cells from HIF-1α- to HIF-2α-dependent signaling promoting stem cell characteristics, aggressive tumor growth and invasion. Cancer Res 2011; 71: 4015–4027. 21512133 10.1158/0008-5472.CAN-10-4142 PMC3268651

[bib21] Mole DR, Blancher C, Copley RR, Pollard PJ, Gleadle JM, Ragoussis J et al. Genome-wide association of hypoxia-inducible factor (HIF)-1alpha and HIF-2alpha DNA binding with expression profiling of hypoxia-inducible transcripts. J Biol Chem 2009; 284: 16767–16775. 19386601 10.1074/jbc.M901790200 PMC2719312

[bib22] Fraisl P, Mazzone M, Schmidt T, Carmeliet P. Regulation of angiogenesis by oxygen and metabolism. Dev Cell 2009; 16: 167–179. 19217420 10.1016/j.devcel.2009.01.003

[bib23] Tang N, Wang L, Esko J, Giordano FJ, Huang Y, Gerber HP. Loss of HIF-1alpha in endothelial cells disrupts a hypoxia-driven VEGF autocrine loop necessary for tumorigenesis. Cancer Cell 2004; 6: 485–495. 15542432 10.1016/j.ccr.2004.09.026

[bib24] Florczyk U, Czauderna S, Stachurska A, Tertil M, Nowak W, Kozakowska M. Opposite effects of HIF-1α and HIF-2α on the regulation of IL-8 expression in endothelial cells. Free Radic Biol Med 2011; 51: 1882–1892. 21925595 10.1016/j.freeradbiomed.2011.08.023 PMC3202637

[bib25] Xia G, Kageyama Y, Hayashi T, Kawakami S, Yoshida M, Kihara K. Regulation of vascular endothelial growth factor transcription by endothelial PAS domain protein 1 (EPAS1) and possible involvement of EPAS1 in the angiogenesis of renal cell carcinoma. Cancer 2001; 91: 1429–1436. 11301389 10.1002/1097-0142(20010415)91:8<1429::aid-cncr1149>3.0.co;2-v

[bib26] Akeno N, Czyzyk-Krzeska MF, Gross TS, Clemens TL. Hypoxia induces vascular endothelial growth factor gene transcription in human osteoblast-like cells through the hypoxia-inducible factor 2alpha. Endocrinology 2001; 142: 959–962. 11159870 10.1210/endo.142.2.8112

[bib27] Rankin EB, Rha J, Unger TL, Wu CH, Shutt HP, Johnson RS et al. Hypoxia- inducible factor-2 regulates vascular tumorigenesis in mice. Oncogene 2008; 27: 5354–5358. 18490920 10.1038/onc.2008.160 PMC2575082

[bib28] Imamura T, Kikuchi H, Herraiz MT, Park DY, Mizukami Y, Mino-Kenduson M et al. HIF-1α and HIF-2α have divergent roles in colon cancer. Int J Cancer 2009; 124: 763–771. 19030186 10.1002/ijc.24032 PMC2682346

[bib29] Maranchie JK, Vasselli JR, Riss J, Bonifacino JS, Linehan WM, Klausner RD. The contribution of VHL substrate binding and HIF1-alpha to the phenotype of VHL loss in renal cell carcinoma. Cancer Cell 2002; 1: 247–255. 12086861 10.1016/s1535-6108(02)00044-2

[bib30] Raval RR, Lau KW, Tran MG, Sowter HM, Mandriota SJ, Li JL et al. Contrasting properties of hypoxia-inducible factor 1 (HIF-1) and HIF-2 in von Hippel-Lindau-associated renal cell carcinoma. Mol Cell Biol 2005; 25: 5675–5686. 15964822 10.1128/MCB.25.13.5675-5686.2005 PMC1157001

[bib31] Kondo K, Klco J, Nakamura E, Lechpammer M, Kaelin WG Jr. Inhibition of HIF is necessary for tumor suppression by the von Hippel-Lindau protein. Cancer Cell 2002; 1: 237–246. 12086860 10.1016/s1535-6108(02)00043-0

[bib32] Kondo K, Kim WY, Lechpammer M, Kaelin WG Jr. Inhibition of HIF2alpha is sufficient to suppress pVHL-defective tumor growth. PLoS Biol 2003; 1: E83. 14691554 10.1371/journal.pbio.0000083 PMC300692

[bib33] Mandriota SJ, Turner KJ, Davies DR, Murray PG, Morgan NV, Sowter HM et al. HIF activation identifies early lesions in VHL kidneys: evidence for site-specific tumor suppressor function in the nephron. Cancer Cell 2002; 1: 459–468. 12124175 10.1016/s1535-6108(02)00071-5

[bib34] Yang SL, Liu LP, Jiang JX, Xiong ZF, He QJ, Wu C. The correlation of expression levels of HIF-1α and HIF-2α in hepatocellular carcinoma with capsular invasion, portal vein tumor thrombi and patients' clinical outcome. Jpn J Clin Oncol 2014; 44: 159–167. 24374892 10.1093/jjco/hyt194

[bib35] Covello KL, Simon MC, Keith B. Targeted replacement of hypoxia-inducible factor-1alpha by a hypoxia-inducible factor-2alpha knock-in allele promotes tumor growth. Cancer Res 2005; 65: 2277–2286. 15781641 10.1158/0008-5472.CAN-04-3246

[bib36] Branco-Price C, Zhang N, Schnelle M, Evans C, Katschinski DM, Liao D et al. Endothelial cell HIF-1α and HIF-2α differentially regulate metastatic success. Cancer Cell 2012; 21: 52–65. 22264788 10.1016/j.ccr.2011.11.017 PMC3334270

[bib37] Mazumdar J, Hickey MM, Pant DK, Durham AC, Sweet-Cordero A, Vachani A et al. HIF-2alpha deletion promotes Kras-driven lung tumor development. Proc Natl Acad Sci USA 2010; 107: 14182–14187. 20660313 10.1073/pnas.1001296107 PMC2922515

[bib38] Chae KS, Kang MJ, Lee JH, Ryu BK, Lee MG, Her NG et al. Opposite functions of HIF-α isoforms in VEGF induction by TGF-β1 under non-hypoxic conditions. Oncogene 2011; 30: 1213–1328. 21057546 10.1038/onc.2010.498

[bib39] Mazumdar J, Dondeti V, Simon MC. Hypoxia-inducible factors in stem cells and cancer. J Cell Mol Med 2009; 13: 4319–4328. 19900215 10.1111/j.1582-4934.2009.00963.x PMC2874971

[bib40] Li Z, Bao S, Wu Q, Wang H, Eyler C, Sathornsumetee S et al. Hypoxia-inducible factors regulate tumorigenic capacity of glioma stem cells. Cancer Cell 2009; 15: 501–513. 19477429 10.1016/j.ccr.2009.03.018 PMC2693960

[bib41] Koshiji M, Kageyama Y, Pete EA, Horikawa I, Barrett JC, Huang LE. HIF-1alpha induces cell cycle arrest by functionally counteracting Myc. EMBO J 2004; 23: 1949–1956. 15071503 10.1038/sj.emboj.7600196 PMC404317

[bib42] Koshiji M, To KK, Hammer S, Kumamoto K, Harris AL, Modrich P et al. HIF-1alpha induces genetic instability by transcriptionally downregulating MutSalpha expression. Mol Cell 2005; 77: 793–803. 10.1016/j.molcel.2005.02.015 15780936

[bib43] Gordan JD, Bertout JA, Hu CJ, Diehl JA, Simon MC. HIF-2alpha promotes hypoxic cell proliferation by enhancing c-Myc transcriptional activity. Cancer Cell 2007; 11: 335–347. 17418410 10.1016/j.ccr.2007.02.006 PMC3145406

[bib44] Cummins EP. HIF-2α-a mediator of stem cell altruism? Stem Cell Res Ther 2012; 3: 52. 23253294 10.1186/scrt143 PMC3580482

[bib45] Das B, Bayat-Mokhtari R, Tsui M, Lotfi S, Tsuchida R, Felsher DW. HIF-2α suppresses p53 to enhance the stemness and regenerative potential of human embryonic stem cells. Stem Cells 2012; 30: 1685–1695. 22689594 10.1002/stem.1142 PMC3584519

[bib46] Gan B, Melkoumian ZK, Wu X, Guan KL, Guan JL. Identification of FIP200 interaction with the SC1-TSC2 complex and its role in regulation of cell size control. J Cell Biol 2005; 170: 379–389. 16043512 10.1083/jcb.200411106 PMC2171462

[bib47] Brugarolas J, Lei K, Hurley RL, Manning BD, Reiling JH, Hafen E et al. Regulation of mTOR function in response to hypoxia by REDD1 and the TSC1rrSC2 tumor suppressor complex. Genes Dev 2004; 18: 2893–2904. 15545625 10.1101/gad.1256804 PMC534650

[bib48] De Young MP, Horak P, Sofer A, Sgroi D, Ellisen LW. Hypoxia regulates TSC1/2-mTOR signaling and tumor suppression through REDD1-mediated 14-3-3 shuttling. Genes Dev 2008; 22: 239–251. 18198340 10.1101/gad.1617608 PMC2192757

[bib49] Choi H, Chun YS, Kim TY, Park JW. HIF-2alpha enhances beta-catenin/TCF- driven transcription by interacting with beta-catenin. Cancer Res 2010; 70: 10101–10111. 21159632 10.1158/0008-5472.CAN-10-0505

[bib50] Liu YL, Yu JM, Song XR, Wang XW, Xing LG, Gao BB. Regulation of the chemokine receptor CXCR4 and metastasis by hypoxia-inducible factor in non small cell lung cancer cell lines. Cancer Biol Ther 2006; 5: 1320–1326. 16929169 10.4161/cbt.5.10.3162

[bib51] Winter SC, Shah KA, Han C, Campo L, Turley H, Leek R et al. The relation between hypoxia inducible factor (HIF)-1alpha and HIF-2alpha expression with anemia and outcome in surgically treated head and neck cancer. Cancer 2006; 107: 757–766. 16826581 10.1002/cncr.21983

[bib52] Hui EP, Chan AT, Pezzella F, Turley H, To KF, Poon TC et al. Coexpression of hypoxiainducible factors 1alpha and 2alpha, carbonic anhydrase IX, and vascular endothelial growth factor in nasopharyngeal carcinoma and relationship to survival. Clin Cancer Res 2002; 8: 2595–2604. 12171890

[bib53] Yoshimura H, Dhar DK, Kohno H, Kubota H, Fujii T, Ueda S et al. Prognostic impact of hypoxiainducible factors 1alpha and 2alpha in colorectal cancer patients: correlation with tumor angiogenesis and cyclooxygenase-2 expression. Clin Cancer Res 2004; 10: 8554–8560. 15623639 10.1158/1078-0432.CCR-0946-03

[bib54] Arabi A, Wu S, Ridderstråle K, Bierhoff H, Shiue C, Fatyol K et al. c-Myc associates with ribosomal DNA and activates RNA polymerase I transcription. Nat Cell Biol 2005; 7: 303–310. 15723053 10.1038/ncb1225

[bib55] Li QQ, Sun YP, Ruan CP, Xu XY, Ge JH, He J et al. Cellular prion protein promotes glucose uptake through the Fyn-HIF-2α-Glut1 pathway to support colorectal cancer cell survival. Cancer Sci 2011; 102: 400–406. 21265952 10.1111/j.1349-7006.2010.01811.x

[bib56] Criscimanna A, Duan LJ, Rhodes JA, Fendrich V, Wickline E, Hartman DJ. PanIN-specific regulation of Wnt signaling by HIF2α during early pancreatic tumorigenesis. Cancer Res 2013; 73: 4781–4790. 23749643 10.1158/0008-5472.CAN-13-0566 PMC3736839

[bib57] Sun HX, Xu Y, Yang XR, Wang WM, Bai H, Shi RY et al. Hypoxia inducible factor 2 alpha inhibits hepatocellular carcinoma growth through the transcription factor dimerization partner 3/ E2F transcription factor 1-dependent apoptotic pathway. Hepatology 2013; 57: 1088–1097. 23212661 10.1002/hep.26188 PMC3594482

[bib58] Mignotte B, Vayssiere JL. Mitochondria and apoptosis. Eur J Biochem 1998; 252: 1–15. 9523706 10.1046/j.1432-1327.1998.2520001.x

[bib59] Jurgensmeier JM, Xie Z, Deveraux Q, Ellerby L, Bredesen D, Reed JC. Bax directly induces release of cytochrome c from isolated mitochondria. Proc Natl Acad Sci USA 1998; 95: 4997–5002. 9560217 10.1073/pnas.95.9.4997 PMC20202

[bib60] Menrad H, Werno C, Schmid T, Copanaki E, Deller T, Dehne N et al. Roles of hypoxiainducible factor-1alpha (HIF-1alpha) versus HIF-2alpha in the survival of hepatocellular tumor spheroids. Hepatology 2010; 51: 2183–2192. 20513003 10.1002/hep.23597

[bib61] Zhang H, Bosch-Marce M, Shimoda LA, Tan YS, Baek JH, Wesley JB et al. Mitochondrial autophagy is an HIF-1-dependent adaptive metabolic response to hypoxia. J Biol Chem 2008; 283: 10892–108903. 18281291 10.1074/jbc.M800102200 PMC2447655

[bib62] Mazure NM, Pouysségur J. Atypical BH3-domains of BNIP3 and BNIP3L lead to autophagy in hypoxia. Autophagy 2009; 5: 868–869. 19587545 10.4161/auto.9042

[bib63] Xue X, Shah YM. Hypoxia-inducible factor-2α is essential in activating the COX2/mPGES-1/PGE2 signaling axis in colon cancer. Carcinogenesis 2013; 34: 163–169. 23042097 10.1093/carcin/bgs313 PMC3534191

[bib64] Franovic A, Holterman CE, Payette J, Lee S. Human cancers converge at the HIF-2alpha oncogenic axis. Proc Natl Acad Sci USA 2009; 106: 21306–21311. 19955413 10.1073/pnas.0906432106 PMC2795516

[bib65] Yang H, Li TW, Peng J, Tang X, Ko KS, Xia M et al. A mouse model of cholestasis-associated cholangiocarcinoma and transcription factors involved in progression. Gastroenterology 2011; 141: 378–388. 21440549 10.1053/j.gastro.2011.03.044 PMC3129489

[bib66] Jungermann K, Kietzmann T. Oxygen: modulator of metabolic zonation and disease of the liver. Hepatology 2000; 31: 255–260. 10655244 10.1002/hep.510310201

[bib67] Qu A, Taylor M, Xue X, Matsubara T, Metzger D, Chambon P et al. Hypoxiainducible transcription factor 2alpha promotes steatohepatitis through augmenting lipid accumulation, inflammation, and fibrosis. Hepatology 2011; 54: 472–483. 21538443 10.1002/hep.24400 PMC3145012

[bib68] Gordan JD, Thompson CB, Simon MC. HIF and c-Myc: sibling rivals for control of cancer cell metabolism and proliferation. Cancer Cell 2007; 12: 108–113. 17692803 10.1016/j.ccr.2007.07.006 PMC3215289

[bib69] Scortegagna M, Ding K, Oktay Y, Gaur A, Thurmond F, Yan LJ et al. Multiple organ pathology, metabolic abnormalities and impaired homeostasis of reactive oxygen species in Epas1−/− mice. Nat Genet 2003; 35: 331–340. 14608355 10.1038/ng1266

[bib70] Warburg O. On the origin of cancer cells. Science 1956; 123: 309–314. 13298683 10.1126/science.123.3191.309

[bib71] Shaw RJ. Glucose metabolism and cancer. Curr Opin Cell Biol 2006; 18: 598–608. 17046224 10.1016/j.ceb.2006.10.005

[bib72] Weidemann A, Johnson RS. Biology of HIF-1alpha. Cell Death Differ 2008; 15: 621–627. 18259201 10.1038/cdd.2008.12

[bib73] Bertout JA, Majmundar AJ, Gordan JD, Lam JC, Ditsworth D, Keith B et al. HIF2 alpha inhibition promotes p53 pathway activity, tumor cell death, and radiation responses. Proc Natl Acad Sci USA 2009; 106: 14391–14396. 19706526 10.1073/pnas.0907357106 PMC2726037

[bib74] Chun SY, Johnson C, Washburn JG, Cruz-Correa MR, Dang DT, Dang LH. Oncogenic KRAS modulates mitochondrial metabolism in human colon cancer cells by inducing HIF-1α and HIF-2α target genes. Mol Cancer 2010; 9: 293. 21073737 10.1186/1476-4598-9-293 PMC2999617

[bib75] Lum JJ, Bui T, Gruber M, Gordan JD, DeBerardinis RJ, Covello KL et al. The transcription factor HIF-1a plays a critical role in the growth factor-dependent regulation of both aerobic and anaerobic glycolysis. Genes Dev 2007; 21: 1037–1049. 17437992 10.1101/gad.1529107 PMC1855230

[bib76] Cleven AH, Wouters BG, Schutte B, Spiertz AJ, van Engeland M, de Bruïne AP. Poorer outcome in stromal HIF-2 alpha- and CA9-positive colorectal adenocarcinomas is associated with wild-type TP53 but not with BNIP3 promoter hypermethylation or apoptosis. Br J Cancer 2008; 99: 727–733. 18728663 10.1038/sj.bjc.6604547 PMC2528150

[bib77] Keith B, Johnson RS, Simon MC. HIFlα and HIF2α: sibling rivalry in hypoxic tumour growth and progression. Nat Rev 2012; 12: 9–22. 10.1038/nrc3183 PMC3401912 22169972

[bib78] Zhao D, Zhai B, He C, Tan G, Jiang X, Pan S et al. Upregulation of HIF-2α induced by sorafenib contributes to the resistance by activating the TGF-α/EGFR pathway in hepatocellular carcinoma cells. Cell Signal 2014; 26: 1030–1039. 24486412 10.1016/j.cellsig.2014.01.026

[bib79] Rankin EB, Biju MP, Liu Q, Unger TL, Rha J, Johnson RS et al. Hypoxia-inducible factor-2 (HIF-2) regulates hepatic erythropoietin *in vivo*. J Clin Invest 2007; 117: 1068–1077. 17404621 10.1172/JCI30117 PMC1838939

[bib80] Imai T, Horiuchi A, Wang C, Oka K, Ohira S, Nikaido T et al. Hypoxia attenuates the expression of E-cadherin via up-regulation of SNAIL in ovarian carcinoma cells. Am J Pathol 2003; 163: 1437–1447. 14507651 10.1016/S0002-9440(10)63501-8 PMC1868286

[bib81] Esteban MA, Tran MG, Harten SK, Hill P, Castellanos MC, Chandra A et al. Regulation of Ecadherin expression by VHL and hypoxia-inducible factor. Cancer Res 2006; 66: 3567–3575. 16585181 10.1158/0008-5472.CAN-05-2670

[bib82] Arya M, Ahmed H, Silhi N, Williamson M, Patel HR. Clinical importance and therapeutic implications of the pivotal CXCL12-CXCR4 (chemokine ligand-receptor) interaction in cancer cell migration. Tumour Biol 2007; 28: 123–131. 17510563 10.1159/000102979

[bib83] Ceradini DJ, Kulkarni AR, Callaghan MJ, Tepper OM, Bastidas N, Kleinman ME et al. Progenitor cell trafficking is regulated by hypoxic gradients through HIF-1 induction of SDF-1. Nat Med 2004; 10: 858–864. 15235597 10.1038/nm1075

[bib84] Staller P, Sulitkova J, Lisztwan J, Moch H, Oakeley EJ, Krek W. Chemokine receptor CXCR4 downregulated by von Hippel-Lindau tumour suppressor pVHL. Nature 2003; 425: 307–311. 13679920 10.1038/nature01874

[bib85] Erler JT, Bennewith KL, Nicolau M, Dornhofer N, Kong C, Le QT et al. Lysyl oxidase is essential for hypoxia-induced metastasis. Nature 2006; 440: 1222–1226. 16642001 10.1038/nature04695

[bib86] Wang Y, Li Z, Zhang H, Jin H, Sun L, Dong H et al. HIF-1α and HIF-2α correlate with migration and invasion in gastric cancer. Cancer Biol Ther 2010; 15: 376–382. 10.4161/cbt.10.4.12441 20559021

[bib87] Kourakis MI, Giatromanolaki A, Polychronidis A, Simopoulos C, Gatter KC, Harris AL et al. Endogenous markers of hypoxia/anaerobic metabolism and anemia in primary colorectal cancer. Cancer Sci 2006; 97: 582–588. 16827797 10.1111/j.1349-7006.2006.00220.x PMC11159659

[bib88] Ahn YT, Chua MS, Whitlock JPJr, Shin YC, Song WH, Kim Y, Eom CY et al. Rodent-specific hypoxia response elements enhance PAI-1 expression through HIF-1 or HIF-2 in mouse hepatoma cells. Int J Oncol 2010; 37: 1627–1638. 21042733 10.3892/ijo_00000817

[bib89] Li F, Tiede B, Massague J, Kang Y. Beyond tumorigenesis: cancer stem cells in metastasis. Cell Res 2007; 17: 3–14. 17179981 10.1038/sj.cr.7310118

[bib90] Heddleston JM, Li Z, Lathia JD, Bao S, Hjelmeland AB, Rich JN. Hypoxia inducible factors in cancer stem cells. Br J Cancer 2010; 102: 789–795. 20104230 10.1038/sj.bjc.6605551 PMC2833246

[bib91] Keith B, Simon MC. Hypoxia-inducible factors, stem cells, and cancer. Cell 2007; 129: 465–472. 17482542 10.1016/j.cell.2007.04.019 PMC3150586

[bib92] Koh MY, Powis G. Passing the baton: the HIF switch. Trends Biochem Sci 2012; 37: 364–472. 22818162 10.1016/j.tibs.2012.06.004 PMC3433036

[bib93] Salnikov AV, Liu L, Platen M, Gladkich J, Salnikova O, Ryschich E et al. Hypoxia induces EMT in low and highly aggressive pancreatic tumor cells but only cells with cancer stem cell characteristics acquire pronounced migratory potential. PLoS One 2012; 7: e46391. 23050024 10.1371/journal.pone.0046391 PMC3458836

[bib94] Wilson WR, Hay MP. Targeting hypoxia in cancer therapy. Nat Rev Cancer 2011; 11: 393–410. 21606941 10.1038/nrc3064

[bib95] Rankin EB, Giaccia AJ. The role of hypoxia-inducible factors in tumorigenesis. Cell Death Differ 2008; 15: 678–685. 18259193 10.1038/cdd.2008.21 PMC3050610

[bib96] Wen YA, Stevens PD, Gasser ML, Andrei R, Gao T. Downregulation of PHLPP expression contributes to hypoxia-induced resistance to chemotherapy in colon cancer cells. Mol Cell Biol 2013; 33: 4594–4605. 24061475 10.1128/MCB.00695-13 PMC3838188

[bib97] Burkitt K, Chun SY, Dang DT, Dang LH. Targeting both HIF-1 and HIF-2 in human colon cancer cells improves tumor response to sunitinib treatment. Mol Cancer Ther 2009; 8: 1148–1156. 19435875 10.1158/1535-7163.MCT-08-0944

[bib98] Rapisarda A, Uranchimeg B, Scudiero DA, Selby M, Sausville EA, Shoemaker RH et al. Identification of small molecule inhibitors of hypoxia-inducible factor 1 transcriptional activation pathway. Cancer Res 2002; 62: 4316–4324. 12154035

[bib99] Semenza GL. Defining the role of hypoxia-inducible factor 1 in cancer biology and therapeutics. Oncogene 2010; 29: 625–634. 19946328 10.1038/onc.2009.441 PMC2969168

[bib100] Kong X, Lin Z, Liang D, Fath D, Sang N, Caro J. Histone deacetylase inhibitors induce VHL and ubiquitin-independent proteasomal degradation of hypoxia-inducible factor 1 alpha. Mol Cell Biol 2006; 26: 2019–2028. 16507982 10.1128/MCB.26.6.2019-2028.2006 PMC1430285

[bib101] Qian DZ, Kachhap SK, Collis SJ, Verheul HM, Carducci MA, Atadja P et al. Class II histone deacetylases are associated with VHL-independent regulation of hypoxia-inducible factor 1 alpha. Cancer Res 2006; 66: 8814–8821. 16951198 10.1158/0008-5472.CAN-05-4598

[bib102] Härter M, Thierauch KH, Boyer S, Bhargava A, Ellinghaus P, Beck H et al. Inhibition of hypoxia-induced gene transcription by substituted pyrazolyl oxadiazoles: initial lead generation and structure-activity relationships. ChemMedChem 2014; 9: 61–66. 24285584 10.1002/cmdc.201300357

[bib103] Philips GK, Atkins MB. New agents and new targets for renal cell carcinoma. Am Soc Clin Oncol Educ Book 2014: e222–e227. 24857106 10.14694/EdBook_AM.2014.34.e222

[bib104] Grkovic T, Whitson EL, Rabe DC, Gardella RS, Bottaro DP, Linehan WM et al. Identification and evaluation of soft coral diterpenes as inhibitors of HIF-2α induced gene expression. Bioorg Med Chem Lett 2011; 21: 2113–2115. 21353547 10.1016/j.bmcl.2011.01.127 PMC3061286

[bib105] Rabbani ZN, Spasojevic I, Zhang X, Moeller BJ, Haberle S, Vasquez-Vivar J et al. Antiangiogenic action of redox-modulating Mn(III) meso-tetrakis (Nethylpyridinium-2-yl)porphyrin, MnTE-2-PyP(5+), via suppression of oxidativestress in a mouse model of breast tumor. Free Radic Biol Med 2009; 47: 992–1004. 19591920 10.1016/j.freeradbiomed.2009.07.001 PMC2749298

[bib106] Wang J, Ma Y, Jiang H, Zhu H, Liu L, Sun B et al. Overexpression of von Hippel-Lindau protein synergizes with doxorubicin to suppress hepatocellular carcinoma in mice. J Hepatol 2011; 55: 359–368. 21168458 10.1016/j.jhep.2010.10.043

[bib107] Bhatt RS, Landis DM, Zimmer M, Torregrossa J, Chen S, Sukhatme VP et al. Hypoxia-inducible factor-2alpha: effect on radiation sensitivity and differential regulation by an mTOR inhibitor. BJU Int 2008; 102: 358–363. 18394010 10.1111/j.1464-410X.2008.07558.x PMC4112353

[bib108] Gu YZ, Moran SM, Hogenesch JB, Wartman L, Bradfield CA. Molecular characterization and chromosomal localization of a third alpha-class hypoxia inducible factor subunit HIF3alpha. Gene Expr 1998; 7: 205–213. 9840812 PMC6151950

